# The Integrity of the Corpus Callosum Mitigates the Impact of Blood Pressure on the Ventral Attention Network and Information Processing Speed in Healthy Adults

**DOI:** 10.3389/fnagi.2017.00108

**Published:** 2017-04-24

**Authors:** Nichol M. L. Wong, Ernie Po-Wing Ma, Tatia M. C. Lee

**Affiliations:** ^1^Laboratory of Neuropsychology, The University of Hong KongHong Kong, Hong Kong; ^2^Laboratory of Social Cognitive Affective Neuroscience, The University of Hong KongHong Kong, Hong Kong; ^3^Institute of Clinical Neuropsychology, The University of Hong KongHong Kong, Hong Kong; ^4^The State Key Laboratory of Brain and Cognitive Science, The University of Hong KongHong Kong, Hong Kong

**Keywords:** blood pressure, brain connectivity, cognitive function, aging, MRI

## Abstract

Hypertension is a risk factor for cognitive impairment in older age. However, evidence of the neural basis of the relationship between the deterioration of cognitive function and elevated blood pressure is sparse. Based on previous research, we speculate that variations in brain connectivity are closely related to elevated blood pressure even before the onset of clinical conditions and apparent cognitive decline in individuals over 60 years of age. Forty cognitively healthy adults were recruited. Each received a blood pressure test before and after the cognitive assessment in various domains. Diffusion tensor imaging (DTI) and resting-state functional magnetic resonance imaging (rsfMRI) data were collected. Our findings confirm that elevated blood pressure is associated with brain connectivity variations in cognitively healthy individuals. The integrity of the splenium of the corpus callosum is closely related to individual differences in systolic blood pressure. In particular, elevated systolic blood pressure is related to resting-state ventral attention network (VAN) and information processing speed. Serial mediation analyses have further revealed that lower integrity of the splenium statistically predicts elevated systolic blood pressure, which in turn predicts weakened functional connectivity (FC) within the VAN and eventually poorer processing speed. The current study sheds light on how neural correlates are involved in the impact of elevated blood pressure on cognitive functioning.

## Introduction

Hypertension is highly prevalent in the aging population and the prevalence of it increases with age (Cheng et al., 2009). It is also a risk factor for other health problems, such as stroke (Strandgaard, 1996) and Alzheimer’s disease (AD; Bermejo-Pareja et al., 2010). Furthermore, hypertension has been shown to be related to a broader deterioration of cognitive functions in individuals with AD (Bellew et al., 2004). Hypertension is also associated with poorer cognitive functioning in older adults without clinical conditions (e.g., dementia; Gifford et al., 2013). Hypertensive elderly individuals appear to demonstrate declines in measures of global cognition (Goldstein et al., 2013) as well as in specific domains including working memory (Elias et al., 2010), attention (Hannesdottir et al., 2009) and executive functioning (Waldstein et al., 2005).

The mechanism underlying the relationship between elevated blood pressure and poorer cognitive functions remains unclear, and there is relatively little evidence on how this vascular risk factor provokes brain changes that precede late-life cognitive decline (Knopman et al., 2011; Carmichael, 2014). For instance, increased systolic blood pressure is associated with brain atrophy, reduced gray matter volume and white matter (WM) hyperintensities (Maillard et al., 2012), and increased diastolic blood pressure is associated with brain atrophy (Heijer et al., 2003). At the brain connectivity level, the functional frontoparietal connections are associated with the worsening of cognitive functions in hypertensive individuals, plausibly due to the deficits in WM integrity (Li et al., 2015), such that WM integrity deficits are noticeable in the splenium and are related to poorer global cognition (Gons et al., 2012). Presumably, brain injuries would increase the vulnerability to unhealthy aging, as exemplified by AD (Douaud et al., 2014).

Therefore, in the current study we investigate whether alterations in brain connectivity, accompanied by elevated blood pressure, could be identified before the onset of clinical conditions and apparent cognitive decline. The associations among blood pressure, brain connectivity and cognitive functions in individuals from the normotensive to moderate-severe hypertensive range are explored, using blood pressure measures as continuous metrics. Blood pressure is relatively modifiable (Mensah and Bakris, 2010), and a better understanding of its underlying neural mechanisms would help promote preventive efforts against cognitive decline and would highlight the importance of regulating blood pressure, which is of increasing interest to the general public. Thus, we investigate brain changes associated with elevated blood pressure at the topological and connectivity levels in cognitively healthy individuals aged 60–70 years using diffusion tensor imaging (DTI) and resting-state functional magnetic resonance imaging (rsfMRI). Adults would likely have a drastic increase in systolic and diastolic pressure in this critical period (Franklin et al., 1997).

DTI and rsfMRI provide information on the strength of structural and functional connectivity (FC), respectively. Compared to investigations of WM lesions at the macrostructural level (e.g., Maillard et al., 2012), fewer studies have examined the microstructural properties of WM using DTI. In addition, we also utilize independent components analysis (ICA; Beckmann and Smith, 2004), a whole-brain multivariate approach, to investigate the strength of FC within resting-state frontoparietal functional networks. We focus on the frontoparietal networks because they were found to be affected in hypertensive elderly individuals previously (Li et al., 2015). Different domains of cognitive function are investigated in our sample, including processing speed, working memory, selective and divided attention, visuospatial skills and executive functioning. Negative associations between blood pressure and WM integrity have been reported previously, including in individuals from the normotensive range (Kennedy and Raz, 2009; Leritz et al., 2010), and systolic blood pressure has been reported to be associated with reduced WM integrity in the corpus callosum (e.g., Delano-Wood et al., 2008; Maillard et al., 2012). Therefore, we hypothesize that elevated blood pressure, particularly systolic pressure, is related to reduced WM integrity in the splenium of the corpus callosum and weakened frontoparietal resting-state functional networks. We also hypothesize that frontoparietal functional networks largely explain the impact of elevated blood pressure on cognitive function.

## Materials and Methods

### Participants

A sample of 40 healthy Chinese individuals was recruited from the community (Table 1). They were above 60 years of age and right-handed (Oldfield, 1971), scoring 24 or higher on the Mini Mental State Examination (MMSE; Folstein et al., 1975) and 8 or lower on the Geriatric Depression Scale-short form (GDS; Lesher and Berryhill, 1994). They did not have any history of vascular events (i.e., myocardial infarction, heart failure, stroke, or peripheral vascular disease), any history of cardiac, lung, liver, or renal failure, or any history of neurological or psychiatric disorders. Participants’ blood pressure was measured before and after cognitive assessments. They underwent an MRI scanning session, in which structural T1-weighted, DTI and rsfMRI data were collected. All participants underwent all procedures either within the same day or across different days within the same month. All participants received a $100 supermarket coupon at the end of their participation.

**Table 1:** **Descriptors and cognitive functioning of the subjects**.

Descriptors	Mean	S.D.	Minimum	Maximum
Systolic pressure	137.66	16.804	105.5	174.5
Diastolic pressure	81.01	10.676	64	101
Pulse pressure	56.64	12.953	35	87.5
Hypertensive treatment (Yes:No)	17.23	–	–	–
Age (years)	63.78	2.455	60	70
Gender (M:F)	14.26	–	–	–
Education (years)	10.45	3.544	4	20
MMSE	28.68	1.655	24	30
GDS	1.58	1.678	0	8
Composite score of processing speed	89.20	23.312	46	136
Composite score of working memory	30.05	6.164	18	44
Stroop interference index	1.3913	0.76341	0.1	3.6
CCT interference index	1.202	0.55767	0.2	2.45
JOL score	10.2	0.139	2	15
Total moves used in TOL	34.98	15.556	12	84
Total time spent in TOL	306.84	100.3233	145.9	590

*CCT, Color Trails Test; GDS, Geriatric Depression Scale; JOL, Judgment of Line Orientation; MMSE, Mini Mental State Examination; S.D., standard deviation; TOL, Tower Of London Test*.

This study was approved by the Faculty Ethics Panel of the Faculty of Social Sciences from The University of Hong Kong. Written informed consents were obtained from all participants.

### Blood Pressure Measures

Each participant’s systolic and diastolic brachial blood pressures were measured from their arms using a sphygmomanometer. Pulse pressure was obtained by calculating the difference between systolic and diastolic blood pressure. Every participant’s blood pressure was measured twice (i.e., before and after cognitive assessment), and the average values were used.

### Cognitive Measures

To assess the cognitive functions of our subjects, six domains were measured in the current study: processing speed, working memory, selective attention, divided attention, visuospatial skills and executive functioning.

Processing speed. A composite score of processing speed was obtained by adding the raw scores of the Digit Symbol Coding and Symbol Search according to the Wechsler Adult Intelligent Scale Third Edition—Taiwanese version (WAIS-III; Wechsler, 1997).

Working memory. A composite score of working memory was obtained by adding the raw scores of the Arithmetic and Digit Span according to the WAIS-III (Wechsler, 1997).

Selective attention. A validated Chinese translation of the Victoria version of the Stroop Color and Word Test (SCWT) was used (Lee and Chan, 2000). An interference index was obtained by subtracting each participant’s reaction time in the incongruent color-word condition (C) from that in the color-dots condition (D) and dividing the result by their reaction time in C.

Divided attention. The Color Trails Test (CTT) comprising Parts A and B was used (D’Elia et al., 1996). Part A required participants to join the digits in ascending order and Part B required participants to join the digits in ascending order with alternating colors. An interference index was obtained by subtracting each participant’s reaction time in Part A from that in Part B and dividing the result by their reaction time in Part A.

Visuospatial skills. The 15-item short form of Judgment of Line Orientation (JLO) was used, comprising Form V items in the following order: 16, 9, 6, 2, 12, 30, 7, 17, 19, 28, 20, 21, 26, 24 and 22 (Qualls et al., 2000).

Executive functioning. The Tower of London test (TOL; Culbertson and Zillmer, 1999) was used with 10 problems of increasing complexity. Participants had a maximum of 2 min and 20 moves to solve each problem. Their total number of moves and the total time were recorded for analysis.

### MRI Data Acquisition and Preprocessing

All participants’ MRI data were acquired via a Philips 3T scanner with a standard 8-channel head coil. DTI data were acquired with TE = 65 ms, TR = 9426 ms, NEX = 2, flip angle = 90°, FOV = 225 × 225 mm, voxel size = 1.56 × 1.56 × 2 mm, one *b* = 0 reference and 32 *b* = 1000 diffusion directions and axial acquisition. rsfMRI data were acquired with TE = 30 ms, TR = 3000 ms, flip angle = 90°, FOV = 230 × 230 mm, voxel size = 2.88 × 2.88 × 4 mm, 160 volumes and axial acquisition.

**Figure 1:**
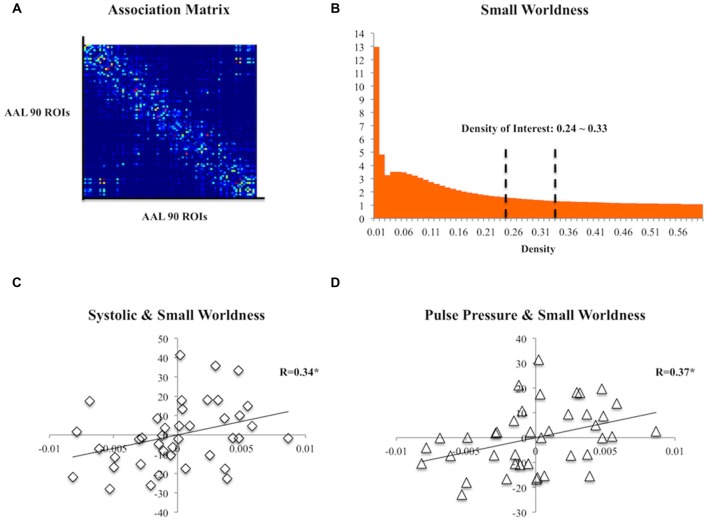
**(A)** A group association matrix computed from 90 regions of interest (ROIs) from the Automated Anatomical Labeling (AAL) atlas.** (B)** The mean Small Worldness (SW) of the subjects at different threshold density with the density range of our interest specified. **(C)** Scatter plot of residuals of systolic pressure and the area under the curve (AUC) of SW after regressing age, gender and education. **(D)** Scatter plot of residuals of pulse pressure and the AUC of SW after regressing age, gender and education. *p < 0.05.

The DTI data were first corrected for eddy current and motion distortions using FSL (FMRIB, Oxford, UK) and were then entered into the DSI studio^1^ for tensor fitting and deterministic tractography using whole-brain seeding with fiber count = 100,000, turning angle = 60°, fractional anisotropy (FA) >0.13 threshold, step size = 0.78, minimum length = 10 and maximum length = 500. Automated Anatomical Labeling (AAL) atlas (Tzourio-Mazoyer et al., 2002) was non-linearly registered to each subject’s diffusion space and each subject’s association matrix was then computed according to the 90 regions defined by the AAL atlas (i.e., cerebellum excluded). The number of streamlines between any pairs of regions was defined as strength of edges (Figure 1A). Using the Matlab-based package GAT (Hosseini et al., 2012) and the Small Worldness (SW; Humphries and Gurney, 2008), a measure of brain topology, was obtained for each matrix at a range of threshold densities between 0.24 and 0.33 with steps of 0.1 (Figure 1B). The minimum density was chosen so that none of the networks were fragmented, whereas the maximum density was chosen to avoid random networks (i.e., not random when SW >1), which are less likely to represent a biologically valid network (Kaiser and Hilgetag, 2006). The SW captures the trade-off between local clustering and path length and any changes in the index might indicate a shift in the balance of network segregation and integration (Rubinov and Sporns, 2010). The area under the curve (AUC; Ginestet et al., 2011) of the SW within the defined threshold density range of each association matrix was then calculated for topological analysis.

The DTI data that were corrected for eddy current and motion distortions were also entered into the FSL to obtain FA maps. Tract-Based Spatial Statistics (TBSS; Smith et al., 2006) implemented in the FSL were used. All of the FA maps were aligned to the default FMRIB58_FA template and were transformed to the Montreal Neurological Institute (MNI) standard space via a nonlinear registration. A mean FA image generated from the sample was produced and thinned to compute a mean FA skeleton with a threshold of FA >0.2. All subjects’ FA maps were projected onto the mean FA skeleton for voxel-wise analyses of integrity within the main tracts (i.e., the skeleton).

**Figure 2:**
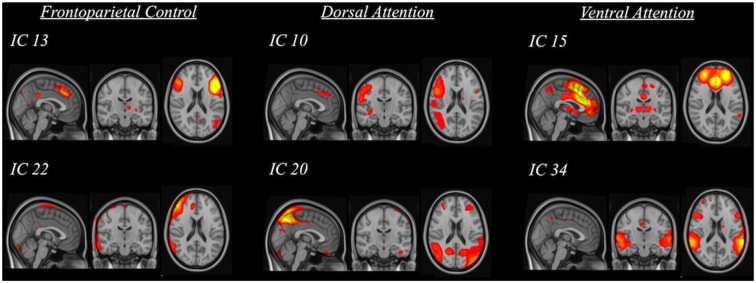
**Spatial maps of significant independent components (IC) that were grouped into three categories based on their relation to standard frontoparietal resting-state networks (i.e., frontoparietal control, dorsal attention and ventral attention)**.

The rsfMRI data were preprocessed with a whole-brain multivariate approach using MELODIC^2^ implemented in the FSL. The first 10 volumes of each subject’s data were first removed to account for signal stabilization. The data were then motion corrected, slice-timing corrected and skull-stripped. They were spatially normalized to the 4-mm MNI standard space with 12° of freedom via co-registering to their skull-stripped T1-weighted images with 6° of freedom. Finally, the data were smoothed with 5 mm FWHM and were high-pass filtered at 100 s. ICA was used to decompose the data into various spatiotemporal independent components (IC; i.e., 42 components were found). The spatial map of each IC was compared to three reference resting-state networks (Yeo et al., 2011) using cross-correlations to identify the IC of our sample that had significant spatial similarity (i.e., *R* > 0.264) with the three resting-state frontoparietal networks (i.e., frontoparietal control, dorsal attention and ventral attention; Figure 2). The significant threshold was determined using the table of critical values for Pearson’s *r* with the corresponding degree of freedom. Significant ICs were then used to yield subject-specific FC maps through dual regression (Beckmann et al., 2009) in the FSL. This procedure involves regressing the component on each subject’s data to obtain subject-specific time series first and then regressing the subject-specific time series on each subject’s data again to obtain subject-specific connectivity map for each significant IC corresponding to the three resting-state networks. These final subject-specific connectivity maps were used for voxel-wise analysis of the FC between specific clusters of voxels and the resting-state network components.

### Statistical Analysis

To rule out possible pre-post changes in blood pressure measures, repeated measures of analysis of covariance (ANCOVA) were performed on the blood pressure measures controlling for age, gender and education. Partial correlations between blood pressure measures and SW were investigated, controlling for age, gender and education. A general linear model (GLM) approach was used to identify how blood pressure measures could be related to brain connectivity at the voxel level. Specifically, systolic, diastolic, or pulse pressure was entered as a regressor, together with age, gender and education as covariate regressors on all subjects’ FA maps masked by the mean FA skeleton, or resting-state FC data masked by the significant IC. Using the randomize function in FSL, permutation tests based on 5000 permutations were performed with a 0.05 significance level after correcting for family-wise error (FWE) using threshold-free cluster enhancement (TFCE). To further correct for multiple testing of the components involved in the resting-state frontoparietal networks, a significance level of 0.005 was adopted for analyzing FC. The region of interest (ROI) approach was then applied and the mean values of regional FA and FC within significant clusters of the corresponding contrast were retrieved and partially correlated with the cognitive measures—including composite score of processing speed, composite score of working memory, interference index of SCWT, interference index of CTT, visuospatial performance on JLO and executive functioning captured by TOL—controlling for age, gender and education. Moderation analyses were performed to examine whether the associations between FA or FC and cognitive measures were dependent on the blood pressure measures. A parallel mediation model was then established to examine how brain variables might mediate the relationship between blood pressure measures and cognitive function in the subjects. Finally, serial mediation analyses were performed based on the findings from the moderation and parallel mediation analyses. Moderation and mediation analyses were performed using PROCESS macro^3^ in SPSS v20.0 with 1000 bootstrap samples (Hayes, 2013). The significance of the mediation paths in the mediation model was based on the inferences from the 95% bias-corrected confidence interval (CI). The percent mediation was also reported as an effect size measure for the indirect effects in the mediation analyses.

## Results

### Blood Pressure and Sample Characteristics

Blood pressure measures did not differ between pre- and post-assessment (systolic: *F*_(1,36)_ = 0.042, *p* = 0.838; diastolic: *F*_(1,36)_ = 0.166, *p* = 0.686; pulse pressure: *F*_(1,36)_ = 0.000, *p* = 0.991). The average value of each measure was used for the subsequent analyses.

Among all subjects, 17 of them were on drugs for hypertension treatment. The average duration was 124 months (minimum = 6, maximum = 360), and no significant age difference between those on drugs and others was detected (*F*_(1,36)_ = 0.005, *p* = 0.943).

None of the blood pressure measures were related to age, years of education, or scores of MMSE or GDS (all *p* values > 0.05). They did not correlate with scores of MMSE and GDS even when controlling for age, gender and years of education. Systolic pressure was related to diastolic and pulse pressure positively, controlling for age, gender and years of education (systolic and diastolic: *R*_(35)_ = 0.642, *p* < 0.001; systolic and pulse pressure: *R*_(35)_ = 0.804, *p* < 0.001; diastolic and pulse pressure: *R*_(35)_ = 0.060, *p* = 0.724).

Subjects’ composite processing-speed scores were negatively correlated to their total time spent in TOL (*R*_(35)_ = −0.606, *p* < 0.001). Their composite scores of working memory were positively correlated with their performance in JLO (*R*_(35)_ = 0.392, *p* = 0.016). Their total moves and total time spent in TOL were correlated with each other (*R*_(35)_ = 0.409, *p* = 0.012).

### Blood Pressure Relates to Cognitive Function

Both systolic (*R*_(35)_ = −0.334, *p* = 0.044) and pulse pressure (*R*_(35)_ = −0.337, *p* = 0.041) were negatively correlated with the composite score of the processing speed. Trending associations were observed in systolic pressure with the Stroop interference index (*R*_(35)_ = −0.293, *p* = 0.079) and with the total time spent in the TOL (*R*_(35)_ = 0.315, *p* = 0.057). Trending associations were also observed between pulse pressure and total moves (*R*_(35)_ = 0.281, *p* = 0.093) and with the total time spent in the TOL (*R*_(35)_ = 0.311, *p* = 0.061). Diastolic pressure did not correlate with any cognitive functions (all *p* values > 0.05), and none of the blood pressure measures were correlated with the composite score of working memory, the CTT interference index, or the visuospatial performance on JLO (all *p* values > 0.05).

### Blood Pressure Relates to Brain Connectivity

At the topological level, both systolic (*R*_(35)_ = 0.34, *p* = 0.04) and pulse pressure (*R*_(35)_ = 0.373, *p* = 0.023) were positively correlated with SW (Figures 1C,D). Diastolic pressure did not correlate with SW (*R*_(35)_ = 0.089, *p* > 0.05).

Structurally, systolic pressure was negatively associated with FA largely in the splenium (*k* = 1295, MNI *x* = −10, *y* = −39, *z* = 15, *T*_peak_ = 5.1) and in the left posterior thalamic radiation (PTR; *k* = 29, MNI *x* = −34, −60, 16, *T*_peak_ = 3.77), whereas diastolic pressure was only negatively associated with the FA in the left PTR (*k* = 272, MNI *x* = −31, *y* = −63, *z* = 15, *T*_peak_ = 4.66). No significant clusters were associated with pulse pressure in the WM along the main tracts.

Functionally, systolic pressure was negatively associated to FC of the right superior temporal gyrus (RSTG) with the ventral attention network (VAN; *k* = 24, MNI *x* = 70, *y* = −26, *z* = 8, *Z*_peak_ = 4.53) and diastolic pressure was negatively associated to the FC of the left STG (LSTG) with the VAN (*k* = 8, MNI *x* = −46, *y* = −34, *z* = 4, *Z*_peak_ = 5.71; Table 2).

**Table 2:** **Brain regions associated with blood pressure**.

ROIs	Measures	Voxels	Peak^a^	MNI *x*	MNI *y*	MNI *z*
**Systolic pressure-related**						
Left SCC	FA	1295	5.1	−10	−39	14
Left FMA	FA	54	3.62	−29	−66	17
Right CP	FA	31	3.78	19	−15	−6
Left PTR	FA	29	3.77	−34	−60	16
Left PTR	FA	6	3.75	−31	−57	13
Right STG in VAN	FC	24	4.53	70	−26	8
**Diastolic pressure-related**						
Left PTR	FA	272	4.66	−31	−63	15
Left STG in VAN	FC	8	5.71	−46	−34	4

*^a^T_peak_ and Z_peak_ values are reported for the FA and the FC respectively. CP, cerebral peduncle; FA, fractional anisotropy; FC, functional connectivity; MNI, Montreal Neurological Institute; PTR, posterior thalamic radiation; ROI, region of interest; STG, superior temporal gyrus; SCC, splenium of corpus callosum*.

### Brain Connectivity Relates to Cognitive Function

Structurally, the FA within the cluster of the left PTR (i.e., related to systolic pressure) was positively correlated with the composite processing-speed scores (*R*_(35)_ = 0.345, *p* = 0.036).

Functionally, the FC of the RSTG with the VAN was positively correlated with the Stroop interference index (*R*_(35)_ = 0.398, *p* = 0.015) and the composite score of processing speed (*R*_(35)_ = 0.359, *p* = 0.029) and negatively correlated with total time spent in the TOL (*R*_(35)_ = −0.342, *p* = 0.038).

### Blood Pressure Moderates the Relationship of Brain Connectivity and Cognitive Function

**Figure 3:**
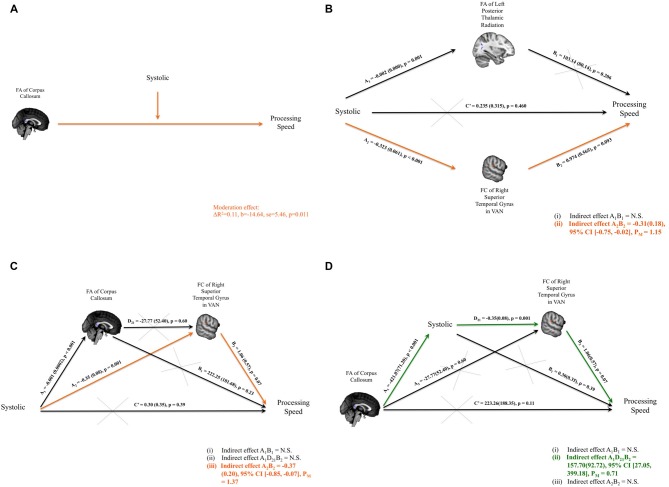
**(A)** Significant interaction between the systolic blood pressure measure and the fractional anisotropy (FA) of corpus callosum on composite scores of processing speed was identified.** (B)** Parallel mediation models were set up to investigate whether FA of the left thalamic radiation and/or FC of right superior temporal gyrus (RSTG) with the ventral attention network (VAN) might mediate the relationship between systolic blood pressure measure and the composite scores of processing speed. Only the mediation path through FC of RSTG with VAN was significant. **(C)** Path model of the FA of the corpus callosum and the FC of RSTG with VAN that mediates the systolic pressure and processing speed. **(D)** Path model of systolic pressure and the FC of the RSTG with VAN that mediates the FA of the splenium of the corpus callosum and processing speed. Significant indirect paths are shaded in orange or green. Structural clusters are shaded in blue and functional clusters are shaded in red. N.S., not significant; P_M_, Percent mediation.

Structurally, the association between FA within the cluster of the splenium of the corpus callosum and composite processing-speed scores was moderated by systolic (Figure 3A), diastolic and pulse pressure (systolic: Δ*R*^2^ = 0.11, *b* = −14.64, SE = 5.46, *p* = 0.011; diastolic: Δ*R*^2^ = 0.03, *b* = −24.52, SE = 10.73, *p* = 0.029; pulse pressure: Δ*R*^2^ = 0.09, *b* = −21.82, SE = 9.08, *p* = 0.022). The association between the FA within the cluster of the left PTR and the composite scores of working memory was moderated by pulse pressure (Δ*R*^2^ = 0.09, *b* = −3.52, SE = 1.55, *p* = 0.030). The association between the FA within the cluster of the splenium of the corpus callosum and the total moves used in TOL was moderated by systolic and pulse pressure (systolic pressure: Δ*R*^2^ = 0.12, *b* = 10.48, SE = 3.72, *p* = 0.008; pulse pressure: Δ*R*^2^ = 0.15, *b* = 19.01, SE = 5.95, *p* = 0.003). The association between the FA within the cluster of the left PTR and the total moves used in TOL was also moderated by pulse pressure (Δ*R*^2^ = 0.08, *b* = 6.83, SE = 3.13, *p* = 0.036). The association between the FA within the cluster of the splenium of the corpus callosum and the total time spent in TOL was moderated by systolic pressure (Δ*R*^2^ = 0.11, *b* = 64.60, SE = 29.03, *p* = 0.033). The association between the FA within the cluster of the splenium of the corpus callosum and the total time used in TOL was moderated by pulse pressure (Δ*R*^2^ = 0.10, *b* = 101.55, SE = 48.10, *p* = 0.042).

Functionally, the association between FC of RSTG with VAN and composite processing-speed scores was moderated by pulse pressure (Δ*R*^2^ = 0.07, *b* = −0.06, SE = 0.03, *p* = 0.043). The association between FC of LSTG with VAN and the Stroop interference index was moderated by pulse pressure (Δ*R*^2^ = 0.09, *b* = −0.002, SE = 0.001, *p* = 0.045). The association between FC of LSTG with VAN and the CTT interference index was moderated by systolic pressure (Δ*R*^2^ = 0.12, *b* = −0.002, SE = 0.001, *p* = 0.028).

### Brain Connectivity Mediates the Relationship between Blood Pressure and Cognitive Function

Systolic pressure was negatively correlated with composite processing-speed scores. It was also significantly related to the FA of the left PTR and the FC of the RSTG with the VAN; and the FA of the left PTR and the FC of the RSTG with the VAN were also associated with composite processing-speed scores. Therefore, mediation analyses were performed to investigate whether the FA of the left PTR and/or the FC of the RSTG with the VAN mediated the association between systolic pressure and composite processing-speed scores. Adopting the parallel mediation model, our results revealed that only FC of the RSTG with the VAN mediated the association between systolic pressure and processing speed (indirect effect = −0.31, SE = 0.18, 95% CI [−0.75, −0.02]; percent mediation = 1.15, SE = 24.55, 95% CI [−2.02, 24.28]; Figure 3B).

### Serial Mediation Analysis

Moderation analysis established that the association between FA within the cluster of the splenium of the corpus callosum and composite processing-speed scores was moderated by systolic pressure. Through parallel mediation analysis, it was found that FC of the RSTG with the VAN mediated the association between systolic pressure and processing speed. Therefore, to investigate how the integrity of the corpus callosum might play a role in the impact of systolic pressure on VAN and processing speed, serial mediation analyses were performed. Two intriguing path models were proposed to investigate whether there are significant indirect paths that denote: (i) the impact of blood pressure (i.e., systolic pressure or pulse pressure) on the link between structural connectivity and FC and processing speed, or (ii) the impact of structural connectivity on the link between blood pressure and FC and processing speed. Importantly, our serial mediation analyses revealed that the lower FA of the splenium of the corpus callosum statistically predicted higher systolic pressure, which in turn allowed for the statistical prediction of weakened FC of the RSTG in the VAN and eventually poorer processing speeds in healthy subjects (indirect effect = 157.70, SE = 92.72, 95% CI [27.05, 399.18]; percent mediation = 0.71, SE = 37.56, 95% CI [0.01, 5.27]). Details of the models that were tested are presented in Figures 3C,D.

## Discussion

Our study provides preliminary evidence that elevated blood pressure is associated with detectable brain connectivity variations in adults without apparent cognitive impairment and that elevated blood pressure is related to slower information processing. To investigate the brain changes that are associated with elevated blood pressure at the topological and connectivity levels, we have investigated SW, WM integrity as captured by FA, and FC within three resting-state frontoparietal networks. In this study, we confirmed our hypothesis that brain changes at the topological (i.e., SW) level and connectivity (i.e., splenium of the corpus callosum and VAN) levels are related to elevated blood pressure. Importantly, the FC of the RSTG with the VAN of cognitively healthy subjects mediates the impact of elevated blood pressure on information-processing speed, with a preserved corpus callosum predicting more favorable blood pressure levels. Together, our results have suggested that the impact of elevated blood pressure on specific cognitive functions and the brain occurs earlier than the onset of clinical conditions.

### Elevated Blood Pressure and Brain Differences

At the topological level, our study identified alterations in the structural topology of the brain in relation to elevated blood pressure. From our behavioral findings, individuals with higher blood pressure tended to have slower information-processing speeds with an upward shift of the SW property of their structural brain networking. SW captures the balance of network segregation and integration via clustering and short paths (Rubinov and Sporns, 2010), and a previous study has reported a similar upward alteration of SW in individuals with AD (He et al., 2008). In view of this, one plausible explanation for the systematic brain changes found in our study is that a disturbance in the networking function of the brain causes it to function at a less than optimal level (Strogatz, 2001). This may partly explain how elevated blood pressure is a high risk factor for developing AD: through its effect on neural mechanisms and brain changes.

Structurally, we observed lower WM integrity of the splenium of the corpus callosum in individuals with higher blood pressure. This parallels another study in the hypertensive elderly (Gons et al., 2012), and this association extends to hypertensive adults (Maillard et al., 2012). Although previous research has demonstrated how WM integrity in multiple fiber bundles could be related to cognitive functioning, our findings align with a previous study that reported that the relationship between variation of blood pressure and WM integrity across the brain might not be as direct and dispersed (Jacobs et al., 2013).

Functionally, connectivity within the frontoparietal networks (i.e., frontoparietal control network [FCN], dorsal attention network [DAN], VAN) was weakened in the elderly with higher blood pressure. The association between weakened frontoparietal network connectivity and higher blood pressure described in our study, parallels the results of a recent study reporting an alteration in the frontoparietal network of hypertensive individuals (Li et al., 2015). Importantly, we did not identify any disrupted structural frontoparietal connectivity relating to blood pressure as suggested in the previous study. This may be due to the fact that the previous study compared brain connectivity in hypertensive individuals to that in normotensive individuals, whereas we see blood pressure measures as a continuous metric and have examined the relationship between blood pressure and brain connectivity.

### Corpus Callosum, Ventral Attention Network and Processing Speed

Based on the mediation analysis, the weakened connectivity of the RSTG in the VAN could largely explain the predictability of high systolic blood pressure on slower information processing speeds in cognitively healthy adults. The frontoparietal system—comprising three subsystems the FCN, the DAN, and the VAN (Yeo et al., 2011)—is important in maintaining mental well-being. The FCN plays a critical role in highly controlled adaptive processes (Cole et al., 2014). The DAN is involved in top-down goal-directed attention to external stimuli, and the VAN specializes in bottom-up stimulus-driven attention to behaviorally relevant, salient or unexpected stimuli (Corbetta and Shulman, 2002). The VAN is a right-lateralized ventral cortical network with the ventral frontal cortex (VFC) and temporoparietal regions as core components. The RSTG has been suggested to be a common site of damage that is related to cognitive problems, such as ventral attentional deficits (Marshall et al., 2002). Specifically, hypertensive individuals were often found to have changes in regional cerebral blood flow within superior temporal cortices (Dai et al., 2008) and over time (Beason-Held et al., 2007), suggesting its vulnerability to raised blood pressure. Variations of the FC of RSTG with the VAN due to elevated blood pressure are likely be related to variations of the bottom-up stimulus-driven attentional controls that would influence their information processing. The importance of good attentional control to maintain efficient processing in older adults is also demonstrated in another behavioral training study (Mackay-Brandt, 2011).

Additionally, the splenium of the corpus callosum is a major interhemispheric commissure with extensive connections linking the visual areas and the parietal and posterior cingulate regions (Knyazeva, 2013) and is closely related to the aging process (Voineskos et al., 2012). From our findings, variation of systolic blood pressure is related to the subject’s processing speed. Moreover, we have found that the association between the WM integrity of the splenium of the corpus callosum and processing speed depends on systolic blood pressure levels. Our serial mediation analyses further identified that the WM integrity in the splenium of the corpus callosum could predict elevated systolic blood pressure and its impact on the VAN and processing speed, but elevated systolic blood pressure could not, in turn, predict the loss of integrity in the splenium of the corpus callosum. Literature might have suggested that higher blood pressure is a risk factor for WM lesions and hyperintensities (Maillard et al., 2012), and our results could have revealed the WM integrity of the splenium of corpus callosum (SCC) as a good indicator of higher blood pressures and its impact. Loss of integrity of the corpus callosum is often related to the aging process (Voineskos et al., 2012), such that adults would likely have higher systolic pressure when they age (Franklin et al., 1997). Alternatively, the corpus callosum has been suggested to play an important role in resilience to stress. For instance, the WM microstructure in the corpus callosum in adults was associated with neuroticism, which could account for their resilience (Xu et al., 2012). The WM integrity of the corpus callosum was also suggested to be sensitive to early-life stress, confirming the implication of the corpus callosum in stress reactivity (Paul et al., 2008). On the other hand, biological response to stress was suggested to lead to raised blood pressure (e.g., Steptoe and Kivimäki, 2012). Drawing these findings together, this one-way statistical prediction might also be interpreted as an indication that the loss of integrity of the corpus callosum during aging (Voineskos et al., 2012) leads to raised blood pressure due to its implication in stress reactivity. It is plausible that other WM tracts (e.g., superior longitudinal fasciculus) are more related to the disruption of the frontoparietal resting-state connectivity due to hypertension (e.g., Li et al., 2015). Prospective studies will need to verify the findings and postulations.

Finally, trending associations were observed in systolic pressure with Stroop interference index and with the total time spent in the TOL. It is plausible that we did not have sufficient statistical power to detect significant correlations. Moreover, reaction time in perceptual and decision tasks is a basic indicator of information processing speed (Anstey et al., 2007). Therefore, as a measure of information processing speed, the composite processing-speed score could more readily be detected and has a more robust relationship with the systolic blood pressure measure. This result also parallels the finding in the study by Singh-Manoux and Marmot (2005).

### Limitations

Our analyses have provided preliminary evidence concerning how blood pressure, structural and FC, and cognitive function might be related to each other on a relatively small sample of adults aged 60–70 years without apparent cognitive decline. We have applied multiple comparisons corrections for voxel-wise analyses to statistically control for errors from multiple comparisons, but the findings should be interpreted with caution and should be regarded as preliminary. Interpretations were made based on correlation, regression and mediation analyses. Owing to the nature of the cross-sectional design, we could not confirm the causality of the relationship without longitudinal data but could only make inferences from the statistical analyses. Therefore, a prospective longitudinal study on the relationship between the brain of cognitively healthy individuals and their blood pressure is needed. Lastly, the blood pressure measures were obtained from participants only before and after the cognitive assessment; the data could be improved by measuring blood pressure multiple times across a period to have a better approximation of participants’ blood pressure levels.

### Conclusions

This study has used a multi-modal neuroimaging approach to investigate how elevated blood pressure may be related to alterations in brain connectivity before the onset of clinical conditions and apparent cognitive decline. Our results have demonstrated the close relationships among elevated blood pressure, changes in brain topology and connectivity, and cognitive function. Although prospective studies are needed to validate our findings, our study has provided findings and shed light on how the functional brain is involved in the impact of elevated blood pressure on cognitive aging.

## Author Contributions

TMCL conceptualized the research idea and planned the study. EP-WM collected the data. NMLW and EP-WM analyzed and interpreted the data. NMLW wrote the manuscript. All authors read and approved the final manuscript. All authors had access to the data, and all authors agreed to submit the article for publication.

## Funding

This study was supported by KKHo International Charitable Foundation and The University of Hong Kong May Endowed Professorship in Neuropsychology awarded to TMCL.

## Conflict of Interest Statement

The authors declare that the research was conducted in the absence of any commercial or financial relationships that could be construed as a potential conflict of interest.

## Footnotes

^1^http://dsi-studio.labsolver.org/

^2^http://fsl.fmrib.ox.ac.uk/fsl/fslwiki/MELODIC

^3^http://www.processmacro.org/
